# The role of red blood cells and cell-free hemoglobin in the pathogenesis of ARDS

**DOI:** 10.1186/s40560-015-0086-3

**Published:** 2015-06-17

**Authors:** David R Janz, Lorraine B Ware

**Affiliations:** Department of Medicine, Section of Pulmonary and Critical Care Medicine, Louisiana State University School of Medicine, New Orleans, LA USA; Department of Medicine, Division of Allergy, Pulmonary, and Critical Care Medicine, Vanderbilt University School of Medicine, T-1218 MCN, 1161 21st Avenue South, Nashville, TN 37232-2650 USA; Department of Pathology, Microbiology, and Immunology, Vanderbilt University School of Medicine, T-1218 MCN, 1161 21st Avenue South, Nashville, TN 37232-2650 USA

**Keywords:** Cell-free hemoglobin, ARDS, Red blood cell, Critical illness, Sepsis

## Abstract

The primary focus of research into the pathophysiology of the acute respiratory distress syndrome (ARDS) has been on the interaction between the lung, underlying causes of ARDS, and the role of white blood cells and platelets in contributing to lung injury. Given a lack of specific therapies for this common complication of critical illness, further insight into the pathophysiology of this syndrome is greatly needed to develop targeted interventions. The red blood cell (RBC) has been reported to undergo deleterious changes in critical illness and be present in the alveoli of patients with ARDS. Release of RBC contents is known to be injurious in other conditions but has only recently been studied in critical illness and ARDS. The contribution of the RBC to ARDS represents a new avenue of research that may produce new, targeted therapies for this deadly syndrome.

## Introduction

The acute respiratory distress syndrome (ARDS) frequently complicates critical illness from a variety of underlying causes [1,2]. This syndrome of acute lung inflammation and non-cardiogenic pulmonary edema is associated with significant morbidity and mortality during the acute hospitalization along with poor long-term outcomes including reduced functional status and increased mortality even beyond the initial hospitalization [3,4]. Although processes of care interventions such as lung protective ventilation [5], prone positioning [6], and neuromuscular blockade [7] may be beneficial, there are no specific pharmacologic interventions to improve outcomes in this patient population. The lack of targeted pharmacologic therapies may be a result of an incomplete understanding of the underlying pathophysiology of ARDS.

Since the first description of ARDS in 1967 [8], investigation into the pathophysiology of ARDS has focused on the interaction between the underlying cause, the lung endothelium, vasculature, and epithelium, and circulating white blood cells and platelets [1]. In the case of direct injury to the lung epithelium by pneumonia, for example, there is an increase in epithelial permeability, and normal clearance of fluid from the alveolus is disrupted [9,10]. The pulmonary capillary endothelium is also disrupted causing an influx of proteinacious fluid and white blood cells into the alveolus resulting in diffuse pulmonary inflammation and coagulation [11]. Given the known inflammation and coagulation that occur with ARDS, pharmacologic therapies have focused on these targets to try to improve clinical outcomes but with no benefit [12-14]. The red blood cell (RBC) also crosses the pulmonary capillary endothelium and can be found in the alveoli of patients with ARDS [1,8,15] but until recently has primarily been viewed as a bystander to the alveolar inflammation and coagulation that occurs in ARDS.

The purpose of this review is to examine the changes that occur to the RBC in critical illness, the contribution of the RBC to alveolar injury in ARDS, and potential interventions for future study.

## Review

### The red blood cell in critical illness

It was first reported over 20 years ago that the RBC cell membrane undergoes changes and is damaged as a result of sepsis, even in the absence of an associated hemolytic condition [16]. The RBC membrane becomes less deformable resulting in damage to the membrane when the RBC transits capillary beds [16,17]. The decreased deformability of the RBC is associated with an increase in systemic oxidative injury and worse clinical outcomes in critical illness including an increase in the number of failing organ systems [16].

Sepsis, the most common cause of indirect ARDS [1], has received the most attention regarding the contribution of systemic illness to damage of the RBC. As a result of the systemic inflammation and coagulation seen in sepsis, there are significant changes in the microcirculation [18], vascular reactivity [19], platelet aggregation, and white blood cell adhesion to the endothelium [18,20]. These changes in the vascular endothelium and interaction between white blood cells and the RBC result in lipid peroxidation of the RBC membrane, alteration of RBC membrane pumps, an influx of calcium into the RBC, and changes in 2,3-diphosphoglycerate levels [21]. All of these contribute to decreased RBC deformability and an increase in RBC aggregation and occlusion of microvascular beds that contribute to organ dysfunction [21].

In addition to occlusion of capillary beds, decreased deformability of RBCs in critical illness also leads to RBC lysis and release of intracellular contents into the circulation. In an experimental model [22], plasma from humans with sepsis was incubated with normal RBCs from healthy volunteers, and changes in the RBC membrane were examined. Exposure of healthy RBCs to plasma from patients with sepsis resulted in binding of annexin V indicative of phosphatidylserine exposure on the outer leaflet, influx of calcium into the RBC leading to RBC aggregation, and an increase in RBC lysis. None of these deleterious effects occurred when normal RBCs were incubated with plasma from non-septic patients. Lysis of RBCs in the intravascular and alveolar spaces results in the release of potentially injurious mediators including cell-free hemoglobin (CFH) that can contribute to organ dysfunction and mortality.

### Cell-free hemoglobin as a mediator of disease

Hemoglobin exists in the red blood cell as a tetramer of globin molecules with ferrous (2+) iron bound to each of the four porphyrin-ring heme groups [23]. During normal RBC turnover in healthy humans, any hemoglobin released from the RBC into the circulation is cleared *via* binding of hemoglobin to haptoglobin leading to CD163 receptor-mediated uptake into the macrophage where hemoglobin is converted to bilirubin by heme oxygenase-1 (HO-1) and ultimately excreted by the liver [24]. Free heme is bound and cleared by binding to hemopexin and macrophage processing by the same mechanisms [24]. Of the hemoglobin processing proteins, HO-1 has received the most attention in past research in critical illness and is known to: (1) Be upregulated in inflammatory and pro-oxidant disease states such as sepsis and ARDS, (2) Be expressed by multiple cell types in the lung, and (3) Decrease pulmonary inflammation and vascular remodeling in animal models of lung injury [25]. Changes in HO-1 levels have been observed in patients with ARDS, asthma, COPD, cystic fibrosis, idiopathic pulmonary fibrosis, and lung cancer, and increased levels of HO-1 are associated with decreased acute lung injury [25].

**Figure 1:**
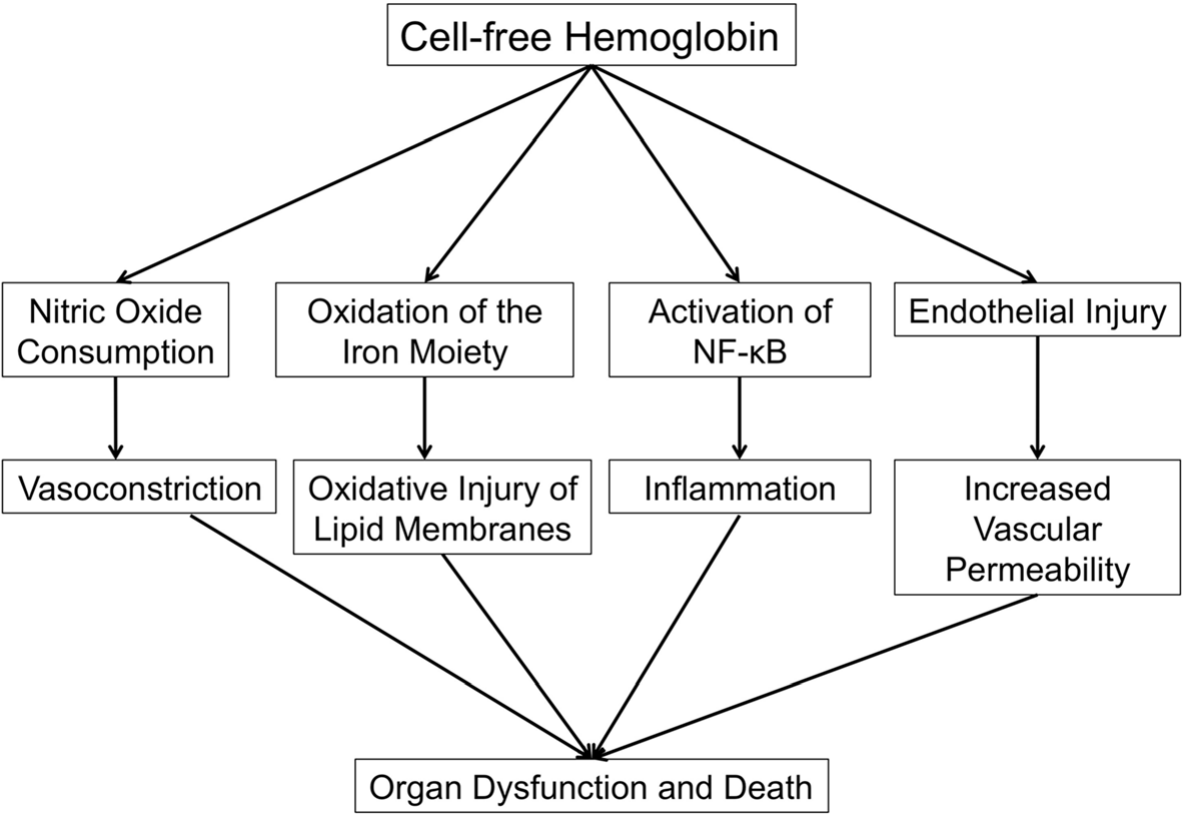
Pathologic mechanisms of the action of plasma cell-free hemoglobin. The pathologic mechanisms by which plasma or air-space cell-free hemoglobin can contribute to organ dysfunction and death in ARDS include nitric oxide consumption, oxidative injury, inflammation, and endothelial injury.

These processes of cell-free hemoglobin (CFH) clearance protect against the four main mechanisms of CFH-mediated organ injury: nitric oxide (NO) consumption, endothelial damage, inflammation, and oxidative injury (Figure 1). Nitric oxide consumption by CFH only occurs when hemoglobin is released from the RBC into the plasma, and hemoglobin has no significant effect on NO when it is compartmentalized in the red blood cell [26]. In patients with sickle cell disease, a condition characterized by intravascular hemolysis and release of up to 30 grams of CFH from the RBC per day, increases in plasma heme were strongly correlated with an increase in NO consumption and peripheral vasoconstriction which could be reversed with inhaled NO [27]. In a guinea pig model of RBC transfusion, animals transfused with RBCs with a storage duration of 28 days had significantly increased levels of plasma heme compared to animals transfused with RBCs stored for 2 days, and transfusion with RBCs stored for 28 days caused extensive endothelial damage to the vasculature and kidney [28]. Treatment with the CFH scavenger haptoglobin prevented transfusion-induced endothelial damage to both the vasculature and kidney in this model. When alveolar epithelial cells are exposed to CFH, release of inflammatory cytokines occurs *via* the NF-κB pathway, and this inflammatory response is independent of iron oxidation [29]. Finally, when hemoglobin is released from the RBC into an oxidative environment, the ferrous (Fe 2+) iron moiety becomes oxidized to the ferryl (Fe 4+) state, and this ferryl radical is capable of inducing oxidative injury [23]. In an animal model of rhabdomyolysis, characterized by the release of the hemoprotein myoglobin and acute kidney injury, animals with induced rhabdomyolysis had significantly elevated levels of ferryl myoglobin, an increase in oxidative injury as measured by F_2_-Isoprostanes, and an increase in plasma creatinine [30]. Supplementation with acetaminophen, a potent ferryl hemoglobin reductant, prevented the increase in ferryl myoglobin, F_2_-Isoprostanes, and plasma creatinine.

A number of disease states are characterized by the release of CFH from the RBC and include sickle cell anemia [27], hemodialysis [31], cardiac bypass [32], red blood cell transfusion [33], pulmonary arterial hypertension [34], and sepsis [35-37]. In all of these conditions, increased NO consumption, endothelial damage, and oxidative injury by CFH have been associated with poor clinical outcomes including acute kidney injury [32,38,39], myocardial infarction [40], and death [35,40,41]. There has been recent interest in the role of CFH in the pathophysiology of sepsis, the most common cause of ARDS, where CFH can be detected in the plasma of 80% of patients [35,37] and increased levels are associated with an increased risk of in-hospital mortality [35]. Additionally, in experimental animal models of sepsis, blocking the deleterious effects of CFH with the heme-binding protein hemopexin resulted in reduced organ injury and mortality [42].

Compared to other disease states associated with CFH release from RBCs, critically ill patients with sepsis have a unique physiologic state that creates an environment where CFH can induce organ injury due to a “two-hit” set of conditions. First, critically ill patients, including those with sepsis, are known to have a reduced capacity to detoxify CFH. Decreased levels of haptoglobin, hemopexin, and heme oxygenase-1 occur in critical illness, including patients with sepsis and ARDS, and decreased levels have been associated with poor clinical outcomes [25,43]. Second, this reduced capacity to detoxify CFH is combined with the oxidative environment that occurs in critical illness [44] that can cause oxidation of the iron moiety of CFH from the ferrous (2+) to the ferric (3+) and the ferryl (4+) state [23]. Both ferric hemoglobin (methemoglobin) and the ferryl hemoglobin radical can be injurious [29]. The ferryl radical is capable of causing organ injury *via* peroxidation of lipid membranes. Products of lipid peroxidation, including F_2_-Isoprostanes and Isofurans, can be detected in critically ill patients and are associated with organ dysfunction [35,44].

### Cell-free hemoglobin and ARDS

Alveolar hemorrhage was noted in the first description of ARDS [8]; however, until recently, the presence of RBCs in the alveoli has been viewed primarily as a marker of alveolar capillary barrier permeability and disease severity rather than a pathologic mediator of injury. The oxidative environment and lack of hemoglobin processing proteins in the alveoli during ARDS [1,15,25] create an ideal environment for RBCs that have traversed the pulmonary endothelium and epithelium to cause injury. In animal studies of ARDS, RBCs are known to break down and release CFH into the alveoli; air-space levels of CFH are associated with the severity of acute lung injury and increased markers of lipid peroxidation [15]. Additionally, instillation of RBCs or CFH into the airways of rats induces acute lung injury [45]. Pulmonary inflammation from instillation of whole blood into the trachea can be attenuated by upregulating the production of haptoglobin, resulting in a decrease in inflammatory cells, alveolar LDH, protein, and macrophage inflammatory protein-2 [46]. Finally, the provision or upregulation of heme oxygenase-1, a protein that is important in the processing of CFH, in animal models of lung injury resulted in reduced lung injury from a variety of injury mechanisms [25,47-50].

An additional mechanism of injury by CFH in ARDS may be mediated specifically by targeted cell surface receptor binding on the alveolar epithelium rather than oxidative injury by the ferryl hemoglobin radical. In an *in vitro* study of human alveolar epithelial cells [29], exposure to methemoglobin (ferric 3+ hemoglobin) stimulated the release of IL-8 and monocyte chemoattractant protein-1 *via* activation of the NF-κB pathway. The supplementation of antioxidants or iron chelators did not alter the effects of methemoglobin, suggesting that CFH can mediate alveolar epithelial injury independent of iron and oxidative pathways.

**Figure 2:**
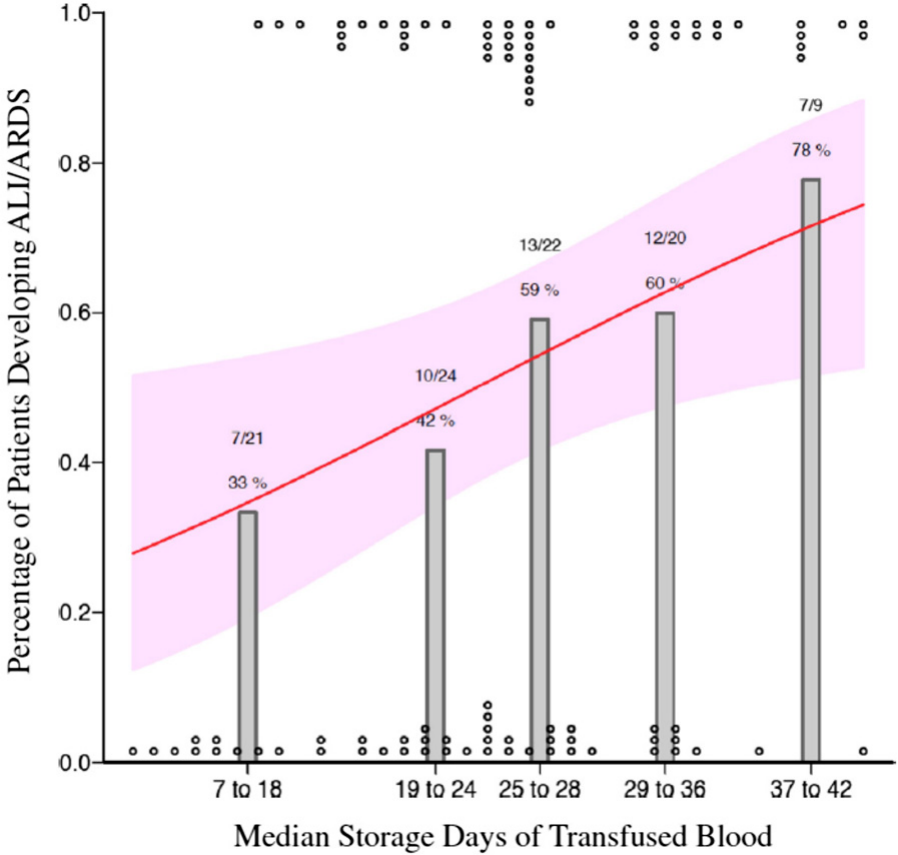
The percentage of patients with sepsis that developed ARDS after transfusion with red blood cells of varying storage duration. A total of 96 critically ill patients with sepsis were analyzed in a retrospective cohort study, all of whom received at least one transfusion of red blood cells. Patients who received red blood cells of a longer storage duration had an associated increased risk of subsequently developing ARDS. *Gray bars* represent the percentage of transfused patients with sepsis who developed ARDS after transfusion. *Circles* at the top and bottom represent individual patients with (top) and without (bottom) ARDS and the median age of transfused red blood cells received. The *red line* and 95% confidence band represent the probability of developing ARDS derived from a multivariable logistic regression model. The patients included in this study did not carry the diagnosis of transfusion-related acute lung injury (TRALI) as they already had a risk factor for ARDS (sepsis), and ARDS that occurred in this study was beyond the 6-hour window for the diagnosis of TRALI. Figure reproduced with permission from SpringerOpen, copyright 2013 [55].

Several lines of evidence suggest that CFH also plays a role in human ARDS. In an observational study of 41 patients with ARDS or hydrostatic pulmonary edema [15], undiluted pulmonary edema samples were obtained *via* endotracheal tube suctioning. Patients with ARDS had significantly higher levels of CFH compared with control patients with hydrostatic pulmonary edema. Also, in patients with diffuse alveolar hemorrhage, alveolar fluid obtained *via* bronchoalveolar lavage showed increasing levels of biomarkers of oxidative injury as the lavage became progressively bloodier. Pulmonary arterial hypertension (PAH) is known to occur during ARDS and is associated with poor outcomes [51,52]. In humans with chronic PAH, plasma CFH is detectable, and increased levels of CFH are associated with increased pulmonary artery pressures, pulmonary vascular resistance, and decreased cardiac index. Additionally, patients with increased plasma CFH had an associated increased risk of PAH-related hospitalization [34]. Whether these findings are relevant to the acute PAH that develops in patients with ARDS is an important topic for future study. Finally, as previously mentioned, transfusion of RBCs, including RBCs of a longer storage duration, results in an increase in plasma CFH and vascular permeability [33,53,54] and is associated with an increased risk of developing ARDS in critically ill patients with sepsis (Figure 2) [55].

### Potential therapeutic interventions

Although we still have a limited understanding of the role of RBCs and CFH in the pathogenesis of ARDS, in other disease states associated with release of CFH, there are therapeutic interventions being developed to improve clinical outcomes. Preliminary studies support a potential benefit of targeting CFH in sickle cell anemia, malaria, sepsis, and other conditions associated with release of CFH. These conditions are more common than ARDS and have some preliminary data regarding the benefit of CFH-inhibition and outcomes. As previously discussed, there are a number of processing proteins required to detoxify CFH to bilirubin including haptoglobin, hemopexin, and heme oxygenase-1 [24]. Animal models of diseases associated with CFH release have focused on supplementation with these proteins to prevent organ dysfunction. In an animal model of RBC transfusion, an intervention known to increase plasma levels of CFH in the recipient, transfusion of RBCs with a long storage duration increased plasma levels of CFH and caused injury to the vascular endothelium and renal injury [28]. Supplementation with haptoglobin completely attenuated the effects of transfusion-induced CFH on the endothelium and kidney. In a separate animal model of sepsis, CFH was found to be elevated and associated with hepatic and renal injury; supplementation with hemopexin to target free heme reversed the effects of CFH in these animals [42]. Finally, supplementation of heme oxygenase-1 to animals with lung injury results in decreased inflammation and alveolar fluid accumulation [25,47,48,50].

Small studies of haptoglobin as a therapeutic CFH scavenger have been conducted in humans with a variety of conditions [56]. In patients undergoing extracorporeal circulation, transfusion with RBCs, or who have acute hemolytic conditions, supplementation with between 3–45 grams of haptoglobin were associated with improved renal function [57-64]; however none of these studies were randomized controlled trials, and some reports included only a single patient receiving haptoglobin as a therapy. Currently, haptoglobin is only available for clinical use in Japan and is not available in the USA.

Acetaminophen has a number of pharmacologic mechanisms, including prostaglandin H_2_ synthase and cyclooxygenase inhibition, that are used to treat pain and fever [65,66]. In addition to these mechanism of action, acetaminophen is a specific ferryl hemoglobin reductant that can inhibit the peroxidase activity of oxidized hemoglobin by reducing the ferryl (4+) hemoglobin radical to the ferric (3+) state [23,66]. In an animal study of rhabdomyolysis where the hemoprotein myoglobin is released into the plasma, animals supplemented with acetaminophen before and after the induction of rhabdomyolysis had reduced oxidative injury as measured by plasma F_2_-Isoprostanes and improved renal function compared with placebo [30]. As previously discussed, extracorporeal circulation of the blood is associated with release of CFH into the plasma, and increased plasma CFH levels are associated with an increased risk of kidney injury [32,67]. In a randomized, placebo-controlled trial of intravenous acetaminophen in pediatric patients undergoing cardiopulmonary bypass, bypass significantly increased CFH, and treatment with intravenous acetaminophen resulted in attenuated oxidative injury compared to placebo [67]. Observational studies of CFH and acetaminophen in critically ill adults with sepsis have shown an association of increased CFH with increased mortality and attenuation of this effect and reduced oxidative injury when patients received acetaminophen as part of their usual care [35]. In a randomized trial of critically ill adults with severe sepsis and detectable plasma CFH, treatment with enteral acetaminophen resulted in a reduction of oxidative injury and improved renal function compared to placebo [36]. As sepsis is the most common underlying cause of ARDS, these findings provide proof of principle that a therapy that targets CFH may be beneficial in ARDS.

No studies to date have examined the efficacy and safety of CFH-targeted therapies such as haptoglobin, hemopexin, heme oxygenase-1, or acetaminophen in humans with ARDS. As we continue to make further insights into the role of the RBC and CFH in the pathogenesis of ARDS, we are likely to see these and other therapies tested in patients with ARDS.

## Conclusions

ARDS is a common and incompletely understood syndrome without targeted therapies. Until recently, investigation into the pathophysiology of ARDS focused on the interaction between white blood cells, platelets, and the pulmonary endothelium and epithelium. The red blood cell, known to be present in the alveoli of patients with ARDS, has previously been viewed as an innocent bystander rather than a major driver of the pathogenesis of ARDS. New insights into the changes that occur in the RBC membrane during critical illness and the role of cell-free hemoglobin in oxidative and endothelial injury have renewed interest in the RBC as a pathologic mediator in ARDS. Further study is needed to better characterize the red blood cell’s role in the pathogenesis of ARDS; however, this line of research is already producing investigation into new therapeutic approaches in ARDS.
